# Association of Upper GI Surgery of Great Britain and Ireland (AUGIS) Delphi consensus recommendations on the adoption of robotic upper GI surgery

**DOI:** 10.1308/rcsann.2024.0014

**Published:** 2024-03-06

**Authors:** PH Pucher, N Maynard, S Body, K Bowling, M Asif Chaudry, M Forshaw, S Hornby, SR Markar, SJ Mercer, SR Preston, B Sgromo, GI van Boxel, JA Gossage

**Affiliations:** ^1^ Portsmouth Hospitals NHS Trust, UK; ^2^ Oxford University Hospitals NHS Trust, UK; ^3^ University Hospitals Dorset NHS Foundation Trust, UK; ^4^ Torbay and South Devon NHS Foundation Trust, UK; ^5^ The Royal Marsden NHS Foundation Trust, UK; ^6^ Glasgow Royal Infirmary, UK; ^7^ Gloucestershire Hospitals NHS Foundation Trust, UK; ^8^ University of Oxford, UK; ^9^ Royal Surrey NHS Foundation Trust, UK; ^10^ Guy’s and St Thomas’ NHS Foundation Trust, UK

**Keywords:** Adoptions, Surgery, Consensuses, Robotic

## Abstract

**Background:**

The adoption of robotic platforms in upper gastrointestinal (GI) surgery is expanding rapidly. The absence of centralised guidance and governance in adoption of new surgical technologies may lead to an increased risk of patient harm.

**Methods:**

Surgeon stakeholders participated in a Delphi consensus process following a national open-invitation in-person meeting on the adoption of robotic upper GI surgery. Consensus agreement was deemed met if >80% agreement was achieved.

**Results:**

Following two rounds of Delphi voting, 25 statements were agreed on covering the training process, governance and good practice for surgeons' adoption in upper GI surgery. One statement failed to achieve consensus.

**Conclusions:**

These recommendations are intended to support surgeons, patients and health systems in the adoption of robotics in upper GI surgery.

## Background

The adoption of robotic or robot-assisted surgery in upper gastrointestinal (GI), or foregut, surgery is expanding rapidly. Implementation is typically being driven by individual centres in partnership with industry providers with little national or specialty society oversight, and without recognised standards for the adoption process. In the past, the unregulated adoption of technological advances has placed patients at risk of harm, such as with the reported rise in rates of bile duct injury at the advent of laparoscopy 30 years ago.^1^ Recent data suggest that, without strong safeguards, the expansion of robotic surgery may similarly lead to increased complications.^2^

In recognition of this fact, the Association of Upper Gastro-Intestinal Surgery of Great Britain and Ireland (AUGIS) set out to establish recommendations for the implementation of robotic upper GI surgery. AUGIS is the leading specialty surgical society in the UK and Ireland, representing surgeons with subspecialist interests including oesophago-gastric cancer, bariatric, benign upper GI, and hepato-pancreato-biliary surgery.

## Methods

A national in-person meeting was held 16 January 2023 in London, UK, to establish the critical questions, problems and considerations associated with the adoption of upper GI robotic surgery. Invitations were circulated to all AUGIS members; attendees were therefore self-selecting surgeons with an interest in robotic surgery and active members of the national upper GI surgical community. Open discussions were led by a steering group and centred on four broad domains: (1) unit/surgeon minimum requirements, (2) case selection, (3) adoption pathway and (4) governance. Discussions and meeting minutes were collated and analysed.

The resulting key questions were considered by a steering group and draft statements prepared, based on best available evidence (where available) and expert opinion. The strength of available evidence was assigned a certainty (i.e. strength of evidence) rating in line with the GRADE framework.^3^

All registered participants from the in-person meeting were subsequently invited to take part in an electronic Delphi process, with statements voted on using a 1–5 Likert scale for disagreement/agreement with each statement, as well as assigning a strength of recommendation. Space for freetext comments was also provided. Consensus was considered achieved if >80% of respondents indicated agreement (Likert rating 4 or 5)^4–6^; strength of recommendation for agreed statements (weak/strong) was based upon a majority vote. Statements that did not achieve consensus were taken forward to a second round of voting.

## Results

A total of 25 questions were developed from the in-person meeting, with resulting statements taken forward to the Delphi process. A total of 35 participants completed both Delphi rounds, including trainees (5/35), and consultants (30/35) with predominant practice in bariatric (6/35), oesophago-gastric (15/35), benign (7/35), and hepato-pancreato-biliary (1/35) surgery. Additionally, 6/35 (17%) respondents were female and 19/35 (54%) currently offered robotic surgery in their routine practice.

Of the 25 statements evaluated by the Delphi consensus group, 24 achieved agreement of >80% in the first round. The one statement that failed to achieve consensus was split into two separate parts and put to a second round of voting. One of the new statements still did not achieve consensus, resulting in 25 statements in total that achieved final consensus, and one that did not (Table 1). For sake of brevity, a narrative summary is provided here. A full discussion of individual statements and associated evidence base is provided in Supplementary Appendix 1.

**Table 1:** Final list of consensus statements

No.	Statement	Strength
1	Stakeholder-backed, society-endorsed, specialty guidelines are crucial to providing endorsement of new techniques and technologies; by considering existing literature and expert opinion, recommendations for the additional resources necessary for surgeons and centres developing an upper GI robotic programme, as well as the internal governance processes which should be followed, will be specified.	Strong
2	AUGIS recognises the need to appropriately disseminate technological advances into broader practice, and supports the adoption of robotic UGI surgery, subject to the recommendations and conditions set out in this guidance. Current evidence for robotic UGI surgery comprises several small randomised trials and predominantly retrospective cohort data. These support the safety of robotic platforms in UGI surgical practice and their non-inferiority compared to existing approaches.	Strong
3	The adoption of robotic surgery requires numerous changes for the surgeon and surgical team, including; competency with a new surgical interface (robotic platform), different instrumentation (robotic graspers and proprietary advanced energy devices), approach (port placement considerations, 4^th^ arm self-assistance), and visualisation (magnified three-dimensional view). A formal training and adoption process is therefore mandatory to ameliorate learning curves and avoid negative impacts on patient outcomes.	Strong
4	In recognition of the known relationship between surgical volume and outcomes, we recommend that individual surgeons should have a minimum of 0.5 robotic all-day operating lists per week, on average. If possible, it is preferable that this number be increased temporarily (and, if necessary, that of trained and experienced colleagues temporarily decreased) during the initial learning phase for new adopters.	Weak
5	We recommend that the minimum following organisational structures be in place for centres adopting robotic surgery: (1) specialty-specific lead to oversee team and specialty-specific governance issues, (2) senior robotic lead to manage access and cross-specialty equality, (3) oversight and approval via a new procedure approvals process, (4) prospective data collection capability, (5) robotic programme-specific internal audit and review processes.	Strong
6	Wherever feasible, we recommend the standardisation of theatre assignments, teams, and staff to build a regular team familiar with each others’ working preferences, to improve efficiency and shorten initial learning curves.	Strong
7	We recommend that surgeons and health systems adopting upper GI robotic surgery should do so with formal input from other specialties within the same organisation which already offer a robotic service, if applicable. Many hospitals will already have an established robotic programme with experienced robotic surgeons in other specialties such as urology, colorectal, or thoracics. Seeking input from experienced surgeons and teams can encourage cross-fertilisation, help establish a governance framework, and provide additional robotic support.	Strong
8	The introduction of upper GI robotic surgery should take place in accordance with local governance processes (such as review by a new procedures committee) to affirm the introduction of robotic surgery within upper GI, and of minimally invasive approaches where transitioning from open surgery.	Strong
9	We recommend that a platform-specific manufacturer-approved curriculum be followed when adopting upper GI robotic surgery. These should include case observation, didactics, dry- and wet-lab training, followed by cadaveric or animal model operating, prior to proceeding to appropriately selected proctored cases.	Strong
10	We recommend that minimum organisational and governance provisions, as set out in this guidance, should be met. Surgeons are expected to complete an approved robotic curriculum and structured proctorship programme.	Strong
11a	We recommend the completion of, and adherence to, agreed adoption pathways and educational curricula when adopting UGI robotic surgery, regardless of the surgeon’s previously preferred approach.	Strong
11b	In order to reduce complexity during the adoption phase, we recommend avoidance of changes to the surgeon’s technique, for example with regard to lymphadenectomy or anastomotic technique, wherever possible. (consensus not achieved, no recommendation made)	None
12	Proctors should be fully trained surgeons experienced in robotic surgery and accredited for the relevant robotic platform. Proctors must have sufficient relevant experience in not only the appropriate specialty but also operation being performed. Minimum case numbers for proctorship must be sufficient to have achieved independent proficiency beyond the learning curve; we recommend no fewer than 50 cases overall, with an appropriate case-mix and volume relative to the cases being proctored.	Weak
13	We recommend that the proctoring process be continued until both proctor and proctee deem the surgery to be safe, independent, and competent. Training should be competency-based, without set minimum or maximum case numbers, with patient safety paramount.	Strong
14	We recommend that units should support two surgeons adopting robotic surgery at a time and that trusts should support dual consultant operating, if desired, especially during the learning curve.	Strong
15	AUGIS supports the adoption of robotic upper GI surgery by all interested surgeons, subject to the conditions set out in these guidelines.	Strong
16	Surgeons should be appropriately supported throughout the adoption of robotic upper GI surgery through educational programmes and proctorship. The ultimate responsibility for ensuring clinical competency and patient outcomes, however, remains the remit of the surgeon and their institution’s governance policies.	Strong
17	Individual training requirements should be completed for each platform.	Strong
18	We recommend a graduated approach to case complexity, with considered case selection (avoidance of particularly challenging patients through disease, frailty, or body habitus) during the initial adoption phase, and competency-based progression (as judged by a qualified proctor or pre-agreed metrics as part of an adoption programme) through complexity levels.	Strong
19	Surgeons must ensure that patients are not placed at risk of suffering a negative outcome, such as increased morbidity, by the adoption of robotic surgery. It is expected that operative times may initially be longer compared to previous non-robotic approaches, and that these will improve over time. Robust internal audit processes should be in place and overseen by local specialty leads.	Strong
20	We recommend that the patient consent process during the adoption phase should include: • More than one documented discussion (e.g. clinic and on day of surgery) • Written as well as verbal explanation of the robotic approach as well as available alternative (non-robotic) surgical approaches • Reason for pursuing a robotic approach, including currently available evidence • Disclosure of surgeon’s experience and learning curve • Consideration of what a reasonable person in the patient’s position might want included in the consent process (in line with Montgomery principles)	Strong
21	Patients should be consented in line with guidance with new or innovative procedures throughout, and beyond the initial adoption phase for robotic surgery. We recommend that this be continued until a minimum case volume of 20 cases in total as primary surgeon is achieved.	Weak
22	Clinical and efficiency outcomes should be prospectively audited and subject to regular review by the local specialty robotic lead.	Strong
23	AUGIS thoroughly supports trainees in the adoption of robotic surgery, though current curricula do not reference robotic training and trainees must prioritise core competencies including laparoscopic and open approaches. We recognise, however, that robotic adoption must take trainees’ needs into consideration to ensure the next generation of surgeons have the technical skills to deliver modern surgical care. Trainees’ access to, and training with, robotic platforms should be supported, subject to availability of resources and trainers.	Strong
24	We support the further study of robotic surgical platforms and its impact on surgical outcomes.	Strong
25	We neither agree nor disagree on the need for further study on the cost effectiveness of robotic vs. laparoscopic surgery, as the pursuit of robotics should be based on improving current and future clinical outcomes. While cost is an important secondary outcome, it is anticipated that further technological maturation and increasing industry competition will continue to drive cost reduction.	Weak

### Consensus-based guidelines

Unregulated adoption of new technologies or techniques, without such guidance, may lead to negative impacts on patient outcome; for example, evidence suggests that the introduction of laparoscopy 30 years ago was associated with a significant rise in rates of bile duct injury during the adoption of laparoscopic cholecystectomy.^1^ AUGIS recognises the need to appropriately disseminate technological advances into broader practice, and supports the adoption of robotic UGI surgery. However, the current evidence base is slim and further study is required.

Patient safety is paramount with any change in surgical practice. It is clear from existing literature that the adoption of robotics is associated with a measurable learning curve. Published data on cholecystectomy,^7,8^ hiatal hernia,^9^ gastrectomy^10^ and oesophagectomy^11^ all observe a measurable change in operative times and/or outcomes over time following initial adoption of robotic surgery. While some evidence suggests that learning curves progress more quickly in robotics than laparoscopy,^12^ there is an ethical and clinical imperative associated with the existence of a learning curve at all: whereas slightly longer operating times for patients operated on early in the adoption phase might be acceptable, any negative impacts on clinical outcomes are not.

No current guidelines for the adoption of robotic upper GI surgery exist. Guidelines for robotic surgery in other specialties, including gynaecology^13^ and urology,^14^ have provided evidence syntheses and made recommendations on the use of robotics in their respective domains; such guidance is lacking in upper GI to date. In Japan in 2010, a patient death following robotic gastrectomy due to pancreatic trauma at the hands of an inexperienced surgeon led to the Japanese Society of Endoscopy Surgery (JSES) making formal recommendations for robotic gastrectomy practice. These included requiring proctorship as one of the conditions for surgeons adoption robotic gastrectomy; however, these recommendations did not address any other areas of robotic surgical upper GI practice.^15^

### Resource and organisational considerations

Current robotic platform manufacturers typically will require minimum robotic access limits as a precondition for investing in surgeon training pathways. These may vary between manufacturer, centre and specialty. However, the well-recognised link between case volumes and outcomes would suggest that regular robotic access should be seen as a requirement for surgeons adoption upper GI robotic surgery, for which we have recommended a minimum of 0.5 lists per week per surgeon; however, there are no specific data regarding adoption of new procedures to support, or refute, this. Wherever feasible, we recommend the standardisation of theatre assignments, teams and staff to build a regular team familiar with each others' working preferences to improve efficiency and shorten initial learning curves.

We recommend further that the minimum following organisational structures be in place for centres adopting robotic surgery: (1) specialty-specific lead to oversee team and specialty-specific governance issues, (2) senior robotic lead to manage access and cross-specialty equality, (3) oversight and approval via a new procedure approvals process, (4) prospective data collection capability and (5) robotic programme-specific internal audit and review processes.

### Adoption and accreditation process

AUGIS is in agreement with a recent US multisociety consensus statement on robotic surgery, which recommends that robotic training curricula may continue to be developed and delivered by respective manufacturers, but that these curricula must be subject to the assessment and approval or appropriate specialty societies.^4^ Specialty-specific robotic training curricula remain somewhat nascent in upper GI surgery but have been developed in other specialties; these consist almost universally of a multimodal training programme including theoretical knowledge training (didactics), case observation, simulation and proctored operating.^16^

Two robotic general surgery platforms are currently in use in the UK: Da Vinci (Intuitive Surgical, Sunnyvale, CA, USA) and Versius (CMR Surgical, Cambridge, UK). Both currently offer appropriate educational curricula suitable for application in upper GI robotic surgery, and at least some educational materials have been developed with, and endorsed by, the Upper GI Robotic Association (UGIRA); as the diversification of providers progresses, we recommend that curricula be assessed on an individual basis for each platform.

UK National Oesophago-Gastric Cancer Audit (NOGCA) data report that 81% of gastrectomies and 49% of oesophagectomies were performed via an entirely open approach (for the years 2019–2021).^17^ Surgeons transitioning to a robotic platform will thus clearly have diverse training backgrounds and experience levels with reference to minimally invasive cancer surgery. This heterogeneity can be overcome with appropriate training and proctorship.^18^ The absence of a training programme, on the other hand, has been associated with negative clinical and oncological outcomes.^11^ The completion of an appropriate robotic curriculum with proficiency-based progression should be considered fundamental to adoption of robotic surgery, regardless of background. The adoption process should be pursued in partnership with an accredited and experienced proctor, until a mutually agreed level of competent independent practice is achieved.

With reference to operative technique, there was disagreement on whether adoption of a robotic platform should change technique, for example, the extent of lymphadenectomy or anastomotic technique for major resections. Those in favour felt that it was important to reduce learning curve complexity by maintaining a familiar operation when learning a new approach in the form of robotic surgery. Others, however, felt that a robotic approach was likely better suited to certain operative techniques and it was important to adapt accordingly, if appropriately proctored. Consensus was not achieved and therefore no recommendation was made in this regard.

We support the simultaneous training of at least two surgeons, with appropriate robotic access provisioned for both. These surgeons might ideally operate together throughout the period of adoption and/or proctorship. Simultaneous training has the benefits of a senior tableside assistant, joint decision-making and increased scheduling flexibility to ensure maximum use of available robotic lists. By having the opportunity to act as both primary surgeon as well as tableside assistant, significant and important understanding of other aspects of the robotic platform may also be gained, such as how to facilitate instrument exchanges, resolve external port or arm clashes and understand ergonomic limitations of the tableside assistant and scrub nurse.

Published literature has suggested a possible link between surgeon factors, such as age, and surgical practices and outcomes.^19–21^ Anecdotally, this has led some to call for training in new surgical technologies and techniques to be left to ‘younger generations’ of surgeons. Evidence to support such a policy is slim and of variable quality. All surgeons who wish to deliver the latest evidence-backed interventional techniques to their patients should be supported to do so appropriately.

### Case selection and outcomes

We recommend a graduated approach to case complexity, with considered case selection (avoidance of particularly challenging patients through disease, frailty or body habitus) during the initial adoption phase, and competency-based progression (as judged by a qualified proctor or pre-agreed metrics as part of an adoption programme) through complexity levels as follows:

• Level 1 cases include cholecystectomy, primary anti-reflux surgery, primary hernia;

• Level 2 cases include paraoesophageal hernias, subtotal gastrectomy;

• Level 3 cases include total gastrectomy, oesophagectomy.

We recommend that the patient consent process during the adoption phase should include:

• More than one documented discussion (e.g. clinic and on day of surgery);
• Written as well as verbal explanation of the robotic approach as well as available alternative (nonrobotic) surgical approaches;
• Reason for pursuing a robotic approach, including currently available evidence;
• Disclosure of surgeon's experience and learning curve;
• Consideration of what a reasonable person in the patient's position might want included in the consent process (in line with Montgomery principles).

Initial operative times are expected to be longer than with established open or laparoscopic approaches, but with appropriate proctorship and patient selection should not otherwise affect clinical outcomes. A robust local system of outcomes monitoring, audit and oversight is recommended.

An enhanced consent process, including additional information about the novelty of the robotic approach in the local institution, comparative evidence for robotic and alternative approaches and the surgeon's experience, should be adhered to until the primary learning curve is overcome.

### Training the next generation of surgeons

Whereas industry-provided training and curricula for upper GI robotic surgery to date have been focused largely on independently practising (consultant) surgeons, training programmes specific to upper GI surgical trainees of varying experience levels are now available, allowing progression from tableside assistant to basic first surgeon procedures. Industry-led programmes to train at resident and fellow level have now also been introduced. AUGIS will continue to support the development of robotic skills and training courses for upper GI trainees. In units with an established robotic programme, trainees should have access to, and training with, robotic platforms.

### Research

Many aspects of upper GI surgery are subject to huge variations in technique that are subject to comparative analysis and research, for example, in terms of fundoplication type in antireflux surgery, to dissection radicality and anastomotic techniques in resectional surgery. The introduction of robotic surgical platforms introduces further variation into the upper GI surgical landscape; we continue to support comparative interventional trials that will support the improvement and optimisation of patient outcomes and surgeon wellbeing.

## Summary

These consensus guidelines provide recommendations to help guide and facilitate the implementation of robotic upper GI surgery in units, developed by UK stakeholders within a framework intended to be broadly applicable to other health systems as well. They have been developed by a self-selected group of surgeons with an interest in robotic surgery via a national open invitation process, and endorsed by the leadership of the UK upper GI national specialty surgical association, AUGIS. They take into account implementation practices, which have been developed overwhelmingly by individual platform manufacturers, but contextualise these within the overarching need for quality standards to be determined by the specialist surgical community rather than industry. This is the only currently available framework to guide the adoption of robotic surgery, to ensure that this advance in surgical technology is introduced in a manner that benefits patients, surgeons and health policy makers alike.

## Conflict of interest statement

S Mercer and G van Boxel are consultants for Intuitive Surgical. No funding or support was received from any source for this work. No other conflicts are declared.
